# A systematic review and meta-analysis: Association between MGMT hypermethylation and the clinicopathological characteristics of non-small-cell lung carcinoma

**DOI:** 10.1038/s41598-018-19949-z

**Published:** 2018-01-23

**Authors:** Lin Chen, Yong Wang, Fen Liu, Liyao Xu, Feifei Peng, Ning Zhao, Biqi Fu, Zijie Zhu, Yu Shi, Jiansheng Liu, Renrui Wu, Chen Wang, Shengmin Yao, Yong Li

**Affiliations:** 1 Department of Internal Neurology, The Affiliated Ganzhou Hospital of Nanchang University (Ganzhou People’s Hospital), Ganzhou, Jiangxi 341000 China; 2 Department of Medical Oncology, The Affiliated Ganzhou Hospital of Nanchang University (Ganzhou People’s Hospital), Ganzhou, Jiangxi 341000 China; 3 Critical Care Medicine, The First Affiliated Hospital of Nanchang University, Nanchan, Jiangxi, 330000 China; 4 Department of Medical Oncology, The First Affiliated Hospital of Nanchang University, Nanchan, Jiangxi, 330000 China; 5 Department of Rheumatology, The First Affiliated Hospital of Nanchang University, Nanchan, Jiangxi, 330000 China

## Abstract

The relationship between O-6-methylguanine-DNA methyltransferase (MGMT) promoter methylation and clinicopathological characteristics of non-small-cell lung carcinoma (NSCLC) has remained controversial and unclear. Therefore, in this study we have undertaken a systematic review and meta-analysis of relevant studies to quantitatively investigate this association. We identified 30 eligible studies investigating 2714 NSCLC patients. The relationship between MGMT hypermethylation and NSCLC was identified based on 20 studies, including 1539 NSCLC patient tissue and 1052 normal and adjacent tissue samples (OR = 4.60, 95% CI = 3.46~6.11, *p* < 0.00001). MGMT methylation varied with ethnicity (caucasian: OR = 4.56, 95% CI = 2.63~7.92, *p* < 0.00001; asian: OR = 5.18, 95% CI = 2.03~13.22, *p* = 0.0006) and control style (autologous: OR = 4.44, 95% CI = 3.32~5.92, *p* < 0.00001; heterogeneous: OR = 9.05, 95% CI = 1.79~45.71, *p* = 0.008). In addition, MGMT methylation was observed to be specifically associated with NSCLC clinical stage, and not with age, sex, smoking, pathological types, and differentiation status. Also MGMT methylation did not impact NSCLC patients survival (HR = 1.32, 95% CI = 0.77~2.28, *p* = 0.31). Our study provided clear evidence about the association of MGMT hypermethylation with increased risk of NSCLC.

## Introduction

Lung cancer has been one of the most common causes of cancer-related death in the world^1^, and its incidence and mortality rates are also much higher than other cancer in China^2^. It consists of two different histological types, non-small-cell lung carcinoma (NSCLC) and small-cell lung carcinoma (SCLC). Among these two, Chinese patients have usually seen higher incidence and mortality rates of NSCLC in the last two decades^3^. Despite some advances in the therapeutic options for NSCLC in recent times, its prognosis is still very poor with a 5-year overall survival^4^. Among the many different reasons for poor outcome, epigenetic modification had played an important role in NSCLC carcinogenesis^5,6^. For instance, it has been seen that DNA methylation typically is associated with silencing the expression of many tumor suppressor genes in the existing cellular pathways^7^. Thus, it requires investigation and identification of specific gene methylation patterns that might be helpful for NSCLC diagnosis and can also act as prognostic markers.

The O-6-methylguanine-DNA methyltransferase (MGMT), a specific DNA damage reversal repairs protein, has been demonstrated to protect tissues against the toxic and carcinogenic effects of alkylating agents by removing adducts from O^6^ position of guanine^8,9^. Epigenetic silencing of MGMT gene by its promoter methylation at specific CpG islands results in loss of its activity in various cancers, including lung cancer^10–12^. However, different studies have shown varying level of MGMT promoter methylation frequency in NSCLC^3,13,14^. But this discrepancy can be attributed to diverse nature of the clinical samples, such as tissues, serum and bronchoalveolar lavage fluid, that were used for this analysis. To be concise, some meta-analysis studies have reported that MGMT methylation is associated with NSCLC incidence^15–17^, but these meta-analysis were based on few studies that involved small number of diverse samples and thus could lead to an erroneous result. Typically, they indicated quite different rates of MGMT hypermethylation from different samples, and only the samples from tumor tissue and plasma showed higher methylation than control group^15,16^. Moreover, these studies have also not thoroughly investigated the relationship between MGMT methylation and clinical characteristics of NSCLC. They only reported the risk between MGMT methylation and NSCLC^15–17^. It is known that different tumor tissue specimens can have some variation, so we in our study have first summarized all published studies which included just the samples from tumor tissues of NSCLC as much as possible, and then performed the systematic review and meta-analysis to quantitatively assess the association of MGMT methylation with incidence and clinical characteristics of NSCLC. With this extensive and careful meta-analysis, we expect to have a better understanding of the role of MGMT methylation in NSCLC.

## Results

### Eligible studies and their characteristics

**Figure 1:**
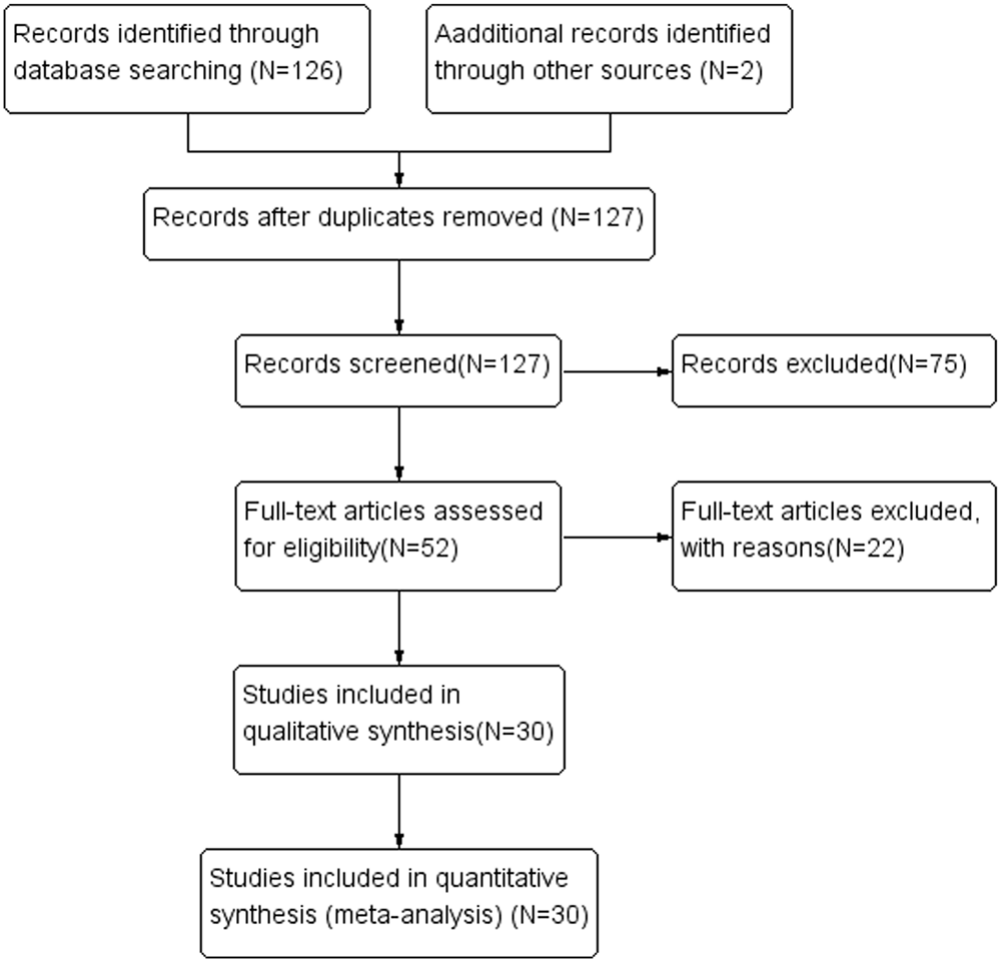
Flow chart depicting the study selection criteria.

Based on the selection criteria, a total of 128 relevant articles were identified. One article was excluded due to duplicative nature, as one another study had similar information. After careful reading of the titles and abstracts, 75 more articles were excluded, as they were either irrelevant or involved just cell or animal studies. The remaining 52 articles were further reviewed in detail, and 22 of them were additionally excluded, as it was observed that tissue specimens were not exclusively were from NSCLC patient’s. In addition, we were unable to extract useful data and some of them were not in English language. Thus, 30 studies^3,11,13,15,18–43^ qualified the required criteria, as shown in Fig. 1, and were assessed in our final meta-analysis. These studies were published between the year 1999 to 2015, and included a total of 2714 NSCLC patients from different countries including, USA, China, Turkey, Germany, Japan, Serbia, Korea, Hong Kong and Taiwan. All these studies detected MGMT DNA methylation by methylation-specific polymerase chain reaction (MSP), Pyrosequencing or Real-Time MSP (RT-MSP) methods. The NOS scores of all studies varied from 6 to 8 points, thereby indicating high quality. Additional basic characteristics of all the included studies have been shown in Table 1.

**Table 1:** Baseline characteristics of eligible studies.

**Study**	**Year**	**Country**	**Ethnicity**	**Type**	**Cases Number**	**Detection Method**	**Methylation site**	**Methylation site N(%)**	**Control style**	**NOS score**
Zöchbauer^13^	2001	USA	Caucasian	NSCLC	107	MSP	Promoter, CpG islands	22(22.56)	A	8
Zhang^3^	2011	China	Asian	NSCLC	78	MSP	Promoter, CpG islands	4(5.13)	A	7
Guo^15^	2015	China	Asian	NSCLC	271	MSP	Promoter, CpG islands	81(29.89)	A	7
Feng^18^	2008	USA	Caucasian	NSCLC	49	MSP	Promoter, CpG islands	6(12.24)	A	7
Guo^11^	2004	USA	Caucasian	NSCLC	20	MSP	Promoter, CpG islands	14(70.00)	A	8
Esteller (1)^19^	1999	USA	Caucasian	NSCLC	22	MSP	Promoter, CpG islands	6(27.27)	A	8
Esteller (2)^20^	1999	USA	Caucasian	NSCLC	34	MSP	Promoter, CpG islands	10(29.41)	A	8
Ekim^21^	2011	Turkey	Caucasian	NSCLC	80	MSP	Promoter, CpG islands	51(63.75)	A	7
Brabender^22^	2003	Germany	Caucasian	NSCLC	90	RT-MSP	Promoter, CpG islands	34(37.78)	A	8
Lin^23^	2009	China	Caucasian	NSCLC	67	MSP	Promoter, CpG islands	1(1.49)	H	7
Yanagawa^24^	2007	Japan	Asian	NSCLC	101	MSP	Promoter, CpG islands	14(13.86)	A	8
Kontic^25^	2012	Serbia	Caucasian	NSCLC	65	MSP	Promoter, CpG islands	8(12.31)	A	7
Kim (1)^26^	2005	Korea	Asian	AC	72	MSP	Promoter, CpG islands	12(16.67)	A	7
Kim (2)^27^	2005	Korea	Asian	NSCLC	61	MSP	Promoter, CpG islands	38(62.30)	A	8
Ishiguro^28^	2013	USA	Caucasian	NSCLC	6	MSP	Promoter, CpG islands	2(33.33)	A	8
Vallböhmer^29^	2006	Germany	Caucasian	NSCLC	91	RT-MSP	Promoter, CpG islands	38(41.76)	A	8
Safar^30^	2005	USA	Caucasian	NSCLC	105	MSP	Promoter, CpG islands	11(10.48)	A	7
Drilon^31^	2014	USA	Caucasian	NSCLC	107	MSP	Promoter, CpG islands	9(8.41)	A	7
Pulling^32^	2003	USA	Caucasian	AC	237	MSP	Promoter, CpG islands	121(51.05)	A	7
Hayashi^33^	2002	Japan	Asian	AC	87	MSP	Promoter, CpG islands	31(35.63)	A	8
Liu^34^	2006	USA	Caucasian	NSCLC	121	MSP	Promoter, CpG islands	37(30.33)	A	7
Chan^35^	2002	Hong Kong	Asian	NSCLC	75	MSP	Promoter, CpG islands	11(14.67)	A	6
Furonaka^36^	2005	Japan	Asian	NSCLC	123	MSP	Promoter, CpG islands	47(38.21)	A	7
Wu^37^	2008	Taiwan	Asian	NSCLC	123	MSP	Promoter, CpG islands	111(50.45)	A	7
Harden^38^	2003	USA	Caucasian	NSCLC	90	RT-MSP	Promoter, CpG islands	14(15.56)	A	8
Buckingham^39^	2010	USA	Caucasian	NSCLC	132	Pyrosequencing	Promoter, CpG islands	14(10.61)	A	7
Topaloglu^40^	2004	USA	Caucasian	NSCLC	31	RT-MSP	Promoter, CpG islands	12(38.71)	A	7
Liu^41^	2010	China	Asian	NSCLC	98	MSP	Promoter, CpG islands	31(31.63)	H	6
Jin^42^	2010	China	Asian	NSCLC	94	MSP	Promoter, CpG islands	16(17.02)	A	6
Kang^43^	2011	China	Asian	NSCLC	77	MSP	Promoter, CpG islands	26(33.77)	H	6

AC: adenocarcinoma; MSP: methylation-specific polymerase chain reaction; RT-MSP: Real-Time MSP; N: number of total; A: autologous control (the control sample from NSCLC themselves); H: heterogeneous control (the control sample from other individuals).

### Correlation analysis between MGMT hypermethylation and NSCLC different clinicopathological features

**Figure 2:**
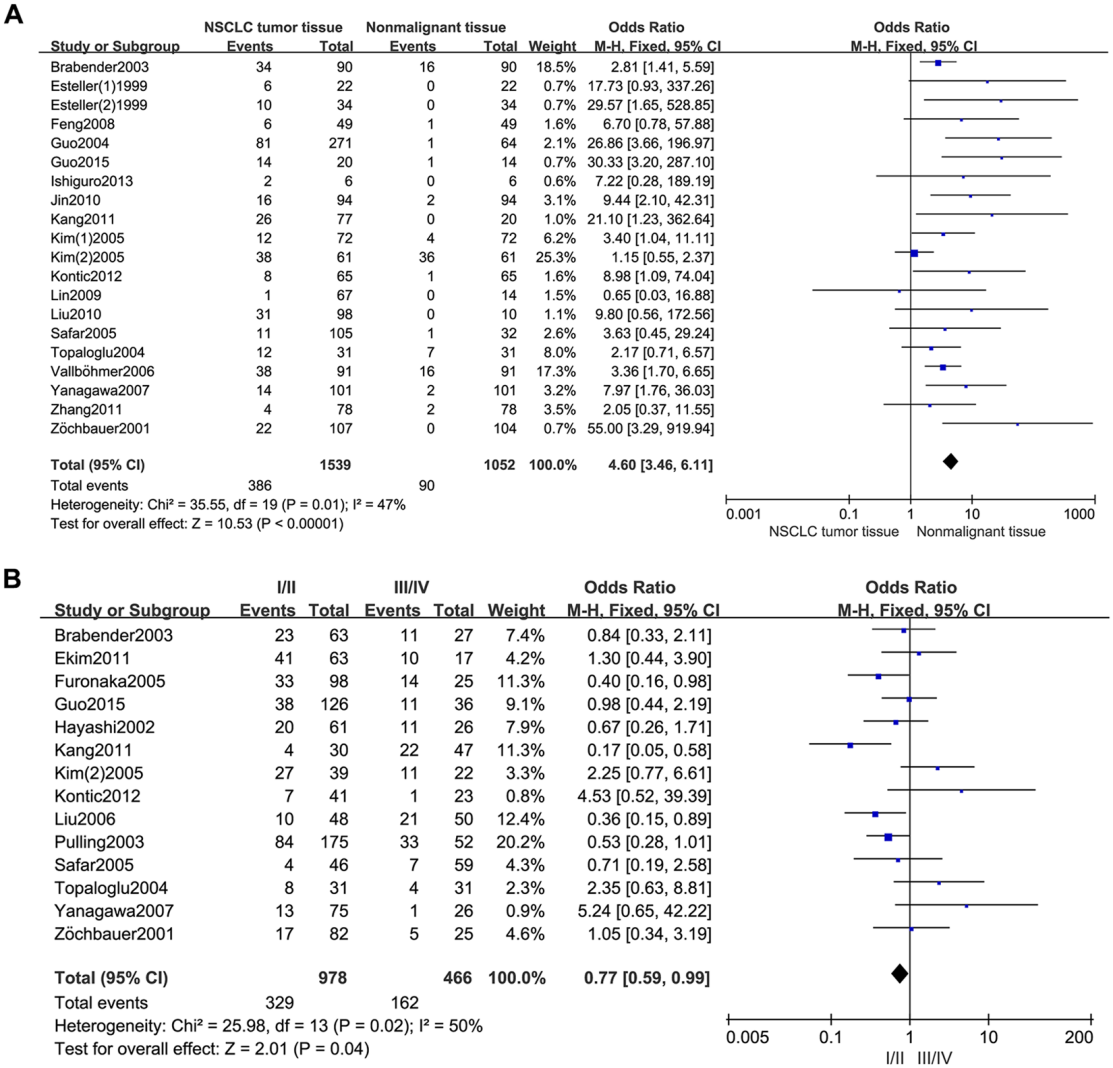
Forest plot representing the meta-analysis of MGMT methylation in NSCLC and clinical stage. (**A**) Forest plot of MGMT methylation in NSCLC tissues verse normal tissues. (**B**) Forest plot showing association of MGMT methylation with clinical stage of NSCLC patients.

**Figure 3:**
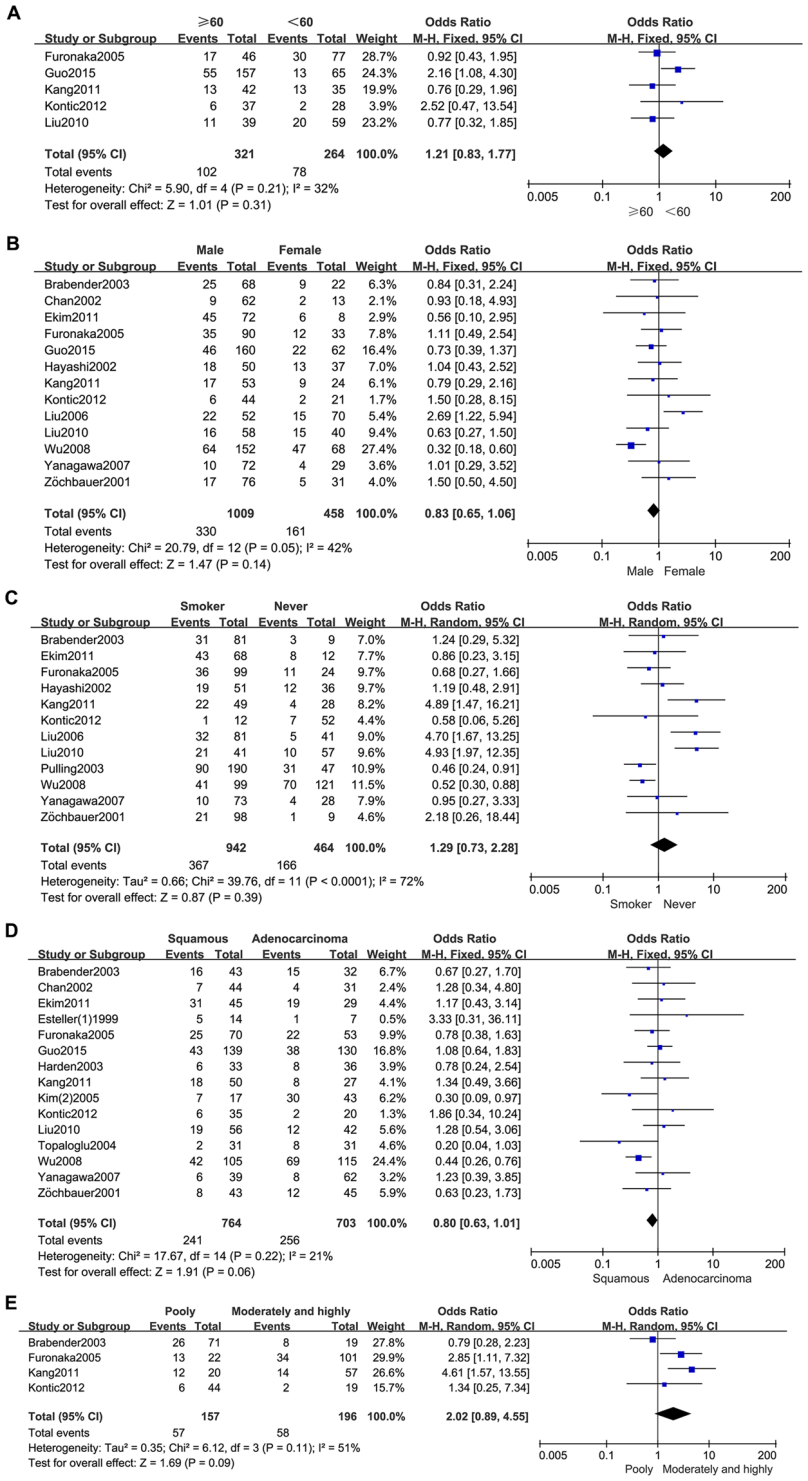
Forest plot representing the meta-analysis of MGMT methylation in clinicopathological features of NSCLC patients. (**A**) Forest plot showing association of MGMT methylation with age status of NSCLC patients. (**B**) Forest plot showing association of MGMT methylation with sex status of NSCLC patients. (**C**) Forest plot showing association of MGMT methylation with smoking status of NSCLC patients. (**D**) Forest plot showing association of MGMT methylation with different pathological types of NSCLC. (**E**) Forest plot showing association of MGMT methylation with differentiation status of NSCLC patients.

**Figure 4:**
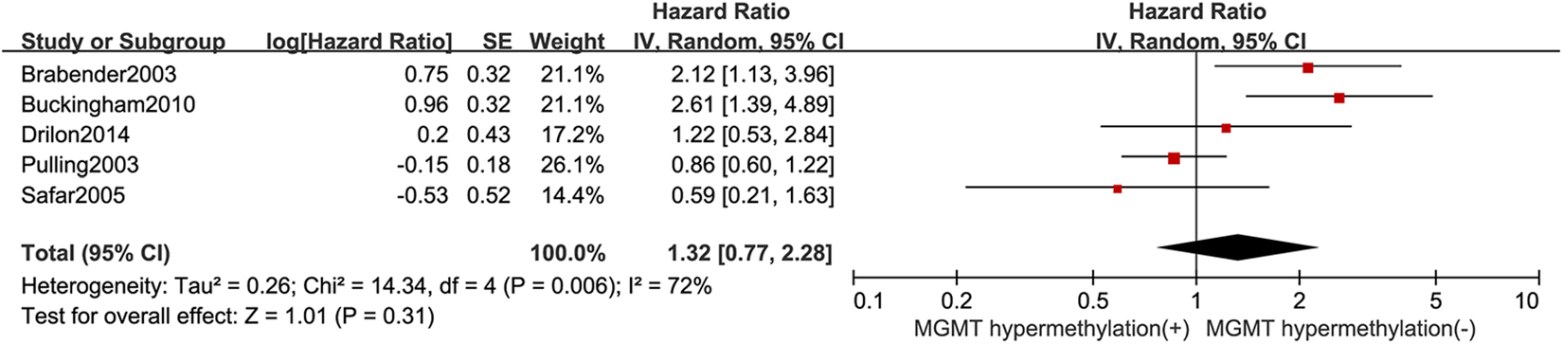
Forest plot showing association of MGMT methylation with overall survival of NSCLC patients.

As shown in Table 2 and Fig. 2, Our analysis revealed that NSCLC tissues had significantly higher MGMT promoter hypermethylation than normal and adjacent tissue samples (OR = 4.60, 95%, CI = 3.46~6.11, *p* < 0.00001, Fig. 2A). The 20 studies including 1539 NSCLC patients’ tissues and 1052 normal and adjacent tissues were involved in the meta-analysis. In an effort to test the correlation between MGMT hypermethylation rate and different staging of NSCLC, we observed that MGMT hypermethylation rate was higher in NSCLC patients with advanced stage than in early stage (OR = 0.77, 95% CI = 0.59~0.99, *p* = 0.04, Fig. 2B). The meta-analysis was assessed based on 14 studies including 466 NSCLC patients with advanced stage and 978 NSCLC patients in early stage. Our meta-analysis were unable to find the correlation between MGMT hypermethylation rate and rest clinicopathological features, including age (OR = 1.21, 95% CI = 0.83~1.77, *p* = 0.31, Fig. 3A), sex (OR = 0.83, 95% CI = 0.65~1.06, *p* = 0.14, Fig. 3B), smoking (OR = 1.29, 95% CI = 0.73~2.28, *p* = 0.39, Fig. 3C), pathological types (OR = 0.80, 95% CI = 0.63~1.01, *p* = 0.06, Fig. 3D), differentiation (OR = 2.02, 95% CI = 0.89~4.55, *p* = 0.09, Fig. 3E). Finally, we also estimated the relationship between overall survival (OS) and the expression of MGMT methylation, by analyzing the data from five observational trails. Four trails were prospective study and one^30^ was retrospective studies. Since there was high heterogeneity (I^2^ = 72%, *p* = 0.006) among these trials, we used random-effects model for statistical adjustment. Our results demonstrated that MGMT hypermethylation in NSCLC did not associate with overall survival (HR = 1.32, 95% CI = 0.77~2.28, *p* = 0.31, Fig. 4).

**Table 2:** Analysis between MGMT hypermethylation and NSCLC different clinicopathological features.

**Analysis**	**Studies N**	**Methylation N(+/**−**)**	**OR(95% CI)**	**Method**	**Heterogeneity**		***P*** **value**
					**I** ^2^ **(%)**	***P*** **value**	
NSCLC	20	476/2115	4.60 [3.46, 6.11]	Fixed	47	0.01	<0.00001
age	5	180/405	1.21 [0.83, 1.77]	Fixed	32	0.21	0.31
sex	13	491/976	0.83 [0.65, 1.06]	Fixed	42	0.05	0.14
smoking	12	533/873	1.29 [0.73, 2.28]	Random	72	<0.00001	0.39
pathological types	15	497/970	0.80 [0.63, 1.01]	Fixed	21	0.22	0.06
differentiation	4	115/238	2.02 [0.89, 4.55]	Random	51	0.11	0.09
clinical stage	14	491/953	0.77 [0.59, 0.99]	Random	50	0.02	0.04

### Subgroup and sensitivity analysis

To investigate the influence of other possible factors on the heterogeneity across studies, we conducted subgroup analysis, according to various confounding factors. Specifically, the patients and controls were stratified based on ethnicity, control style and detection method for subgroup analysis, as shown in Table 3. Our results of NSCLC indicated that MGMT hypermethylation varied with ethnicity (Caucasian: OR = 4.56, 95% CI = 2.63~7.92, *p* < 0.00001; Asian: OR = 5.18, 95% CI = 2.03~13.22, *p* = 0.0006), control style (Auologous: OR = 4.44, 95% CI = 3.32~5.92, *p* < 0.00001; Heterogeneous: OR = 9.05, 95% CI = 1.79~45.71, *p* = 0.008) and detection method (MSP: OR = 6.78, 95% CI = 3.40~13.51, *p* < 0.00001; RT-MSP: OR = 2.91, 95% CI = 1.87~4.53, *p* < 0.00001). I^2^ changed to 27% and 64% in ethnicity subgroup, 50% and 30% in control style subgroup, 53% and 0% in detection method subgroup, compared with 47% of total. The results of subgroup analysis of the association between MGMT hypermethylation and smoking indicated that I^2^ changed to 66% and 80% in ethnicity subgroup, 52% and 0% in control style subgroup, compared with 72% of total. Therefore, the subgroup analysis implied that the factor of control style influence heterogeneity of the association between MGMT hypermethylation and smoking, ethnicity could not explain the heterogeneity.

**Table 3:** Subgroup analysis of the association between MGMT hypermethylation and NSCLC or Smoking.

**Analysis**	**Studies N**	**Patients**	**OR(95% CI)**	**Method**	**Heterogeneity**		***P*** **value**
					**I** ^2^ **(%)**	***P*** **value**	
**NSCLC**							
Ethnicity							
Caucasian	12	1239	4.56 [2.63, 7.92]	Fixed	27	0.18	<0.00001
Asian	8	1352	5.18 [2.03, 13.22]	Random	65	0.005	0.0006
Control style							
Auologous	17	2305	4.44 [3.32, 5.92]	Random	50	0.01	<0.00001
Heterogeneous	3	286	9.05 [1.79, 45.71]	Fixed	30	0.24	0.008
Detection Method							
MSP	17	2167	6.78 [3.40, 13.51]	Random	53	0.006	<0.00001
RT-MSP	3	424	2.91 [1.87, 4.53]	Fixed	0	0.80	<0.00001
Total	20	2591	4.60 [3.46, 6.11]	Fixed	47	0.01	<0.00001
**Smoking**							
Ethnicity							
Caucasian	6	700	1.18 [0.48, 2.92]	Random	66	0.01	0.72
Asian	6	706	1.39 [0.61, 3.16]	Random	80	0.0001	0.43
Control style							
Auologous	10	1231	0.92 [0.57, 1.49]	Random	52	0.03	0.74
Heterogeneous	2	175	4.92 [2.37, 10.19]	Fixed	0	0.99	<0.0001
Total	12	1406	1.29 [0.73, 2.28]	Random	72	<0.00001	0.39

**Figure 5:**
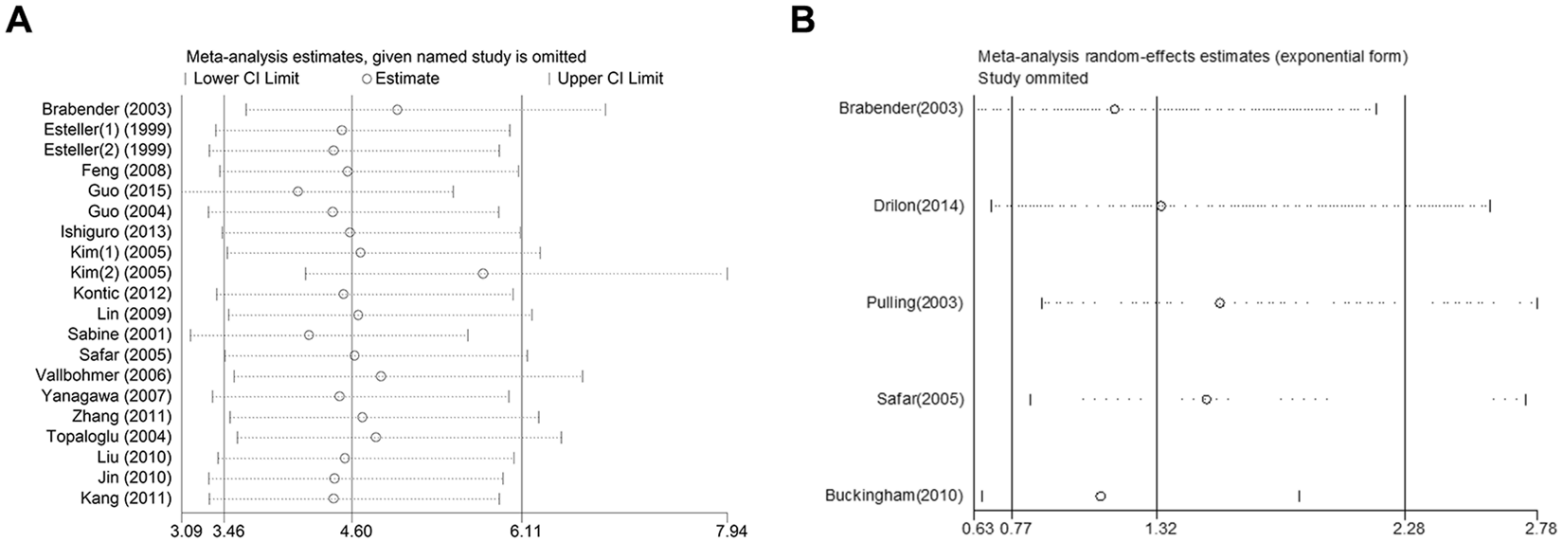
Sensitivity analysis by omitting a single study. (**A**) Sensitivity analysis of the OR coefficients for the association between MGMT methylation and risk of NSCLC. (**B**) Sensitivity analysis of the HR coefficients for the association between MGMT methylation and overall survival of NSCLC patients.

In addition, we also evaluated the sensitivity of our meta-analysis by removing one study at a time and analyzing the remaining studies to assess the stability of the data. The pooled ORs and HR did not significantly alter after the removal of any one study, thereby establishing the stability of our results, as shown in Fig. 5. Interestingly, a moderate heterogeneity was observed (I^2^ = 47%) in the analysis between MGMT hypermethylation and NSCLC, and deletion of one study by Kim (2) *et al*.^33^, significantly reduced the heterogeneity (I^2^ = 25%).

### Publication bias analysis

**Figure 6:**
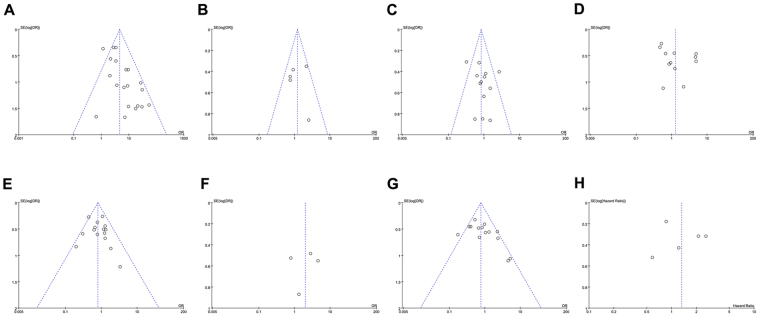
Funnel plots analyses to assess the publication bias between MGMT methylation and different NSCLC clinicopathological characteristics; (**A**) Overall funnel plot from pooled 20 studies, (**B**) Funnel plot based on age, (**C**) Funnel plot based on sex status, (**D**) Funnel plot based on smoking status, (**E**) Funnel plot based on pathological types, **(F**) Funnel plot based on differentiation status, (**G**) Funnel plot based on clinical stage status, and (**H**) Funnel plot based on NSCLC overall survival.

**Figure 7:**
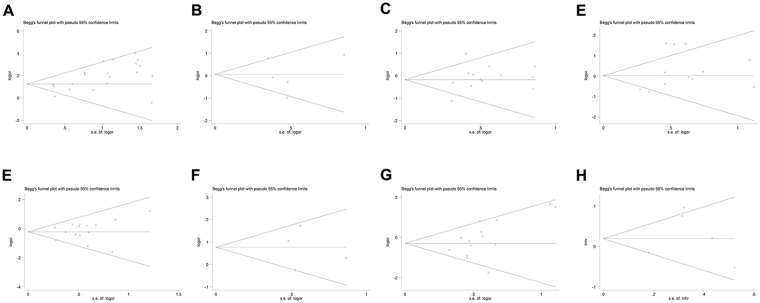
Begg’s funnel plot analyses to assess the publication bias between MGMT methylation and different NSCLC clinicopathological characteristics; (**A**) Overall funnel plot from pooled 20 studies, (**B**) Funnel plot based on age, (**C**) Funnel plot based on sex status, (**D**) Funnel plot based on smoking status, (**E**) Funnel plot based on pathological types, (**F**) Funnel plot based on differentiation status, (**G**) Funnel plot based on clinical stage status, and (**H**) Funnel plot based on NSCLC overall survival.

Further, we also evaluated the publication bias of the selected studies by funnel plot and Begg’s test. If the result of funnel plots showed symmetry and *p* > 0.05 from Begg’s test, indicated that no significant publication bias existed. The funnel plots analysis showed symmetry, as shown in Fig. 6. The Begg’s test also displayed that publication bias was not statistically significant as shown in Fig. 7. These results overall indicated that publication bias had no influence on our meta-analysis.

## Discussion

MGMT is a DNA repair gene located on a human chromosome band 10q26, and encodes a high-efficiency DNA repair protein to protect cells and tissues from disintegration by ubiquitination-dependent proteolysis, by removing alkyl groups from the O6 position of guanine nucleotide^9,44,45^. During the early stage of carcinogenesis, epigenetic and genetic alterations are common events, and silencing of this MGMT gene by its promoter methylation is one of the major mechanism for carcinogenesis in tumor tissues of various cancers, including NSCLC^10,12,46^. In recent years, some studies have reported about MGMT methylation in NSCLC, however the persuasive evidence about its role in NSCLC and clinical significance is not very convincing. Thus, we undertook this meta-analysis to identify the association between MGMT promoter methylation and different clinicopathological characteristics of NSCLC.

Our overall pooled data demonstrated that (1) the frequency of MGMT methylation in NSCLC tissue was much higher than normal tissue samples; (2) MGMT methylation was not correlated with clinicopathological characteristics like age, sex, smoking, pathological types, and differentiated status; (3) MGMT methylation played an important role in the staging and was higher in advanced staged (III and IV) NSCLC tissue than in early staged (I and II) tissue samples; and (4) MGMT methylation could not be a prognostic factor for NSCLC prognosis. MGMT gene promoter methylation is a frequent event in NSCLC tissues showed that the MGMT gene promoter hypermethylation is associated with formation and development of NSCLC. Inactivation of the gene of MGMT play an important role in tumor aberrant progression. Our these observation were consistent with the previously published meta-analyses reports^17,47^, which had also reported similar observations. These findings helped us to speculate that it could be a factor for the NSCLC incidence or its risk. Based on the current technology, DNA methylation patterns of any gene can be detected from all kinds of body fluids. Since DNA methylation is an early event in the tumor initiation and is best-characterized as epigenetic alteration, and has been shown to contribute towards carcinogenesis, it could act as an alternative biomarker^48,49^. In this reference, one can say based on our findings that MGMT methylation can have a potential role for the NSCLC diagnosis. In addition, many other studies have reported that number of methylated genes were associated with the clinical characteristics^3,13,24,34^, however our meta-analyses indicated that MGMT promoter methylation was not associated with any clinical characteristics in NSCLC, except staging. It suggested that the increased ability of proliferation and invasion of NSCLC cells may be associated with MGMT hypermethylation. Inactivation of gene of MGMT could contribute tumor progression. In contrast, the study by Huang *et al*. has reported that MGMT gene methylation was associated with smoking behavior in NSCLC^50^. This different observation can be attributed to less number of studies that were analyzed in that study, as only 8 studies including 817 patients were selected. However, our data is based on the analysis of 12 studies, which included 1304 patients. In addition, similar to our observation of MGMT gene methylation not associated with worse NSCLC survival, the study by Chen *et al*. also confirmed similar results^51^.

Importantly, we observed a moderate heterogeneity (I^2^ = 47%) in our met-analysis. But after deletion of one study conducted by Kim (2) *et al*.^27^, the heterogeneity significantly declined (I^2^ = 25%). Now, it was not very clear about the possible reasons of why the results in their study were so different, but we could speculate that may be the method of MGMT methylation detection was a bit different. As we all know that heterogeneity can be due to different characteristics of the patients, including the ethnicity and control style. To minimize the influence of these confounding factors, we performed subgroup analysis and observed that heterogeneity significantly reduced in several subgroups. High heterogeneity was also observed in smoking (I^2^ = 72%, *p* < 0.0001) and NSCLC overall survival (I^2^ = 72%, *p* = 0.006) meta-analysis. We conducted subgroup analysis and sensitivity analysis in the same way, then we observed that heterogeneity significantly reduced in several subgroups. When using the samples from heterogeneous control, the pooled OR of MGMT methylation in smoking patients was much higher than that in no smoking patients. When using the samples from autologous control, the result was opposite. The reason might be high concentration of MGMT methylation in samples of autologous control. This maybe partly explain the heterogeneity of our study. In sensitivity analysis, the pooled ORs and HR did not significantly alter after the removal of any one study. It suggested the robustness of our results.

However, we have to note that there were some limitations of our meta-analysis. First, our study selection criteria was restrictive to all English language studies only, and it is highly possible that studies published in other language or unpublished data and studies could tilt the overall conclusion. Second, the result could also be influenced by selection and information biases. Negative results were not as conclusive as the positive results. Bias caused by unpublished articles is the same reason for all the meta-analysis. We could collect data as comprehensive as possible. Write letter to the author for their manuscript. The best way to control the publication bias is to register all the clinical studies, and build database. Third, different studies using varying methods to detect the level of MGMT gene methylation can also effect the overall assessment and thus a universal method of methylation detection should be standardized.

In summary, our meta-analysis revealed that MGMT gene methylation was higher in NSCLC tissue samples than normal. Also advanced stage NSCLC patients showed higher methylation than early stage patients. Finally, it would be suffice to say that MGMT methylation is indeed associated with an increased NSCLC risk, and thus has the potential to be a good “biomarker” for NSCLC diagnosis in the future. More importantly, additional large-scale studies would be required to further clarify the value of MGMT methylation in clinical use for NSCLC diagnosis/risk assessment.

## Methods

### Search strategy

We systematically searched the Pubmed, Cochrane library, Embase and China National Knowledge Infrastructure (CNKI) databases to identify the relevant studies between, January 1, 1997 to August 10, 2016. The following search terms were used: (“lung”) and (“cancer” or “tumor” or “neoplasm” or “carcinoma”) and (“methylation”) and (“MGMT” or “O6-methylguanine-DNA methyltransferase gene”). In addition, we also manually searched the reference lists from the relevant retrieved articles and reviews.

### Selection criteria

The studies were selected for the meta-analysis based on the following specific selection criteria. The eligible studies included; (1) NSCLC patients specimens evaluated for MGMT methylation, (2) Studies reporting the relationship between MGMT methylation level and clinicopathological parameters or prognosis in NSCLC patients, (3) studies either having the direct information about the hazard ratio (HR) and 95% confidence interval (CI) for survival, or with a sufficient data where these can be calculated, and (4) studies with definitive detection method of MGMT methylation. However, the studies were excluded if they; (1) were only letters, reviews, editorials, expert opinions, case reports, meeting records or conference abstracts, (2) were not written in English, (3) lacked the information about clinicopathological parameters or sufficient data for the estimation of HR with 95% CI, (4) had NSCLC tissue specimens other than the serum, plasma, pleural effusion, sputum and Bronchoalveolar lavage, (5) were studies conducted on cells or animal only, and (6) were duplicate publications.

### Data extraction

The data from these selected studies was independently extracted and reviewed by two authors, Wang and Chen, according to the predefined criteria from eligible studies. The key characteristics of each study recorded were as follows; first author name, year of publication, country, ethnicity, number of cases, source of sample, MGMT methylation detection method and methylation site and frequency. In addition, the extracted information also included clinicopathological parameters of patients, like age, gender, smoking, histological type of cancer, differentiated status, cancer stage (tumor node metastasis, TNM) and prognosis. All these data for study characteristics and clinical responses have been summarized in a table format.

### Quality assessment

To assure the high quality of our research, all included studies were systematically and independently assessed according to the Newcastle-Ottawa scale (NOS) criteria. The studies were scored by two authors as follows: (1) subject selection, 0~4 points, (2) comparability of subject, 0~2 points, (3) clinical outcome, 0~3 points. The NOS scores ranged from 0 to 9 with a score of ≥7 indicating good quality. All the disagreements were resolved by discussion and consensus with a third author, Yong Li.

### Statistical analysis

Meta-analyses were performed using Review Manager 5.3 (Cochrane Collaboration, Oxford, UK) and STATA 12.0 (Stata Corporation, TX, USA) statistical software. The frequency of MGMT methylation was compared in different clinicopathological parameters and odds ratios (OR), HR and 95% CI were calculated. The pooled OR represented the actual association between MGMT methylation and clinicopathological features. HR implied the hazard of mortality for prognosis (*p* < 0.05 were considered statistically significant). If the value of HR and 95% CI was not directly provided in any study, then we analyzed the K-M curves using Engauge Digitizer version 4.1 software to calculate HR with 95% CI^52^. The heterogeneity among different studies was estimated by the Cochran’s Q test (*p* < 0.05 indicated significant heterogeneity) and I^2^ statistic (0~25%, low heterogeneity; 25~50%, moderate heterogeneity; 50~75%, high heterogeneity; 75~100%, extreme high heterogeneity. According According to the Cochrane handbook, heterogeneity could be accepted if the I^2^ ≤ 50%.)^53,54^. If the I^2^ value was ≥50%, then random effects model was used for meta-analysis, otherwise the fixed effects model was used. Subgroup and sensitivity analysis were performed if a statistically significant heterogeneity was observed in the meta-analysis. The publication bias was assessed by funnel plot^55,56^ and Begg’s test^57^, (*p* < 0.05 indicated significant publication bias).
